# Protective effect of zinc oxide nanoparticles on spinal cord injury

**DOI:** 10.3389/fphar.2022.990586

**Published:** 2022-10-05

**Authors:** Jia Liu, Zhendong Huang, Suhan Yin, Yanping Jiang, Longquan Shao

**Affiliations:** Stomatological Hospital, Southern Medical University, Guangzhou, China

**Keywords:** zinc oxide nanoparticles, spinal cord injury, oxidative stress, PI3K/Akt pathway, cellular apoptosis

## Abstract

The microenvironmental changes in the lesion area of spinal cord injury (SCI) have been extensively studied, but little is known about the whole-body status after injury. We analyzed the peripheral blood RNA-seq samples from 38 SCI and 10 healthy controls, and identified 10 key differentially expressed genes in peripheral blood of patients with SCI. Using these key gene signatures, we constructed a precise and available neural network diagnostic model. More importantly, the altered transcriptome profiles in peripheral blood reflect the similar negative effects after neuronal damage at lesion site. We revealed significant differential alterations in immune and metabolic processes, therein, immune response, oxidative stress, mitochondrial metabolism and cellular apoptosis after SCI were the main features. Natural agents have now been considered as promising candidates to alleviate/cure neuronal damage. In this study, we constructed an *in vitro* neuronal axotomy model to investigate the therapeutic effects of zinc oxide nanoparticles (ZnO NPs). We found that ZnO NPs could act as a neuroprotective agent to reduce oxidative stress levels and finally rescue the neuronal apoptosis after axotomy, where the PI3K-Akt signaling probably be a vital pathway. In conclusion, this study showed altered transcriptome of peripheral blood after SCI, and indicated the neuroprotective effect of ZnO NPs from perspective of oxidative stress, these results may provide new insights for SCI diagnosis and therapeutics.

## Introduction

Spinal cord injury (SCI) is an impairment/trauma of any part of the spinal cord, which can be manifest as a state of spinal shock immediately and result in reversible or irreversible loss of neurological function (Alizadeh et al., 2019). Importantly, SCI can lead to lifelong impairment, including disability and handicap, which can significantly impact the quality of life. However, tissue regeneration and function recovery of SCI is usually limited by the weak intrinsic growth capacity of injured neurons and the hostile environment surrounding the lesions (Anjum et al., 2020). Thus, developing effective strategies for treating SCI has become a research hotspot.

There is a growing awareness that the SCI induced pathophysiological changes may be helpful to assess the injury severity and predict prognosis of patients. Among them, several proteins in the cerebrospinal fluid (CSF) of SCI patients, such as cytokines or other intracellular proteins, have predictive utility as candidate biomarkers for detecting local inflammation and tissue destruction. Comparing to the invasive detection of CSF, the patients’ blood samples could also provide a convenient and valuable alternative. The disruption of the blood-spinal cord barrier following spinal cord trauma means that many of the biomarkers found in CSF are often detectable in serum as well, albeit at lower concentrations. Meanwhile, it has been reported that the circulating immune cells numbers and differentials changes happened after SCI, and these blood-derived immune cells are involved in secondary axonal atrophy and loss, which is likely to reflect the biological features of the evolving lesion. Importantly, inhibition of the infiltration and immune response of these cells at the early stage of injury could reduce the tissue degeneration by attenuating the secondary injury events (Bao et al., 2012; Badner et al., 2016). Therefore, identifying the common biomarkers of the inhibitory microenvironment at the whole body would first provide diagnostic approach after SCI and further provide targets to achieve better therapeutic results. A previous study has shown that the gene expression profile of peripheral blood cells is able to reflect some useful aspects of the whole-body status (Reynés et al., 2018), which could provide an effective strategy to discover the targets for diagnosis and treatment of SCI.

A wide variety of natural agents, including extracts or monomers isolated from plants, animals, microorganisms and minerals, have been applied for prevention or treatment of neurological damage and diseases (Sharifi-Rad et al., 2020). Due to their ability to protect neurons, some specific kinds of natural products have showed potentially optimum effects in SCI model to help the lesion recover and restore the internal homeostasis (Zhang et al., 2016; Tao et al., 2020). Therein, zinc oxide (ZnO), as one of the most widely used metallic oxide materials, occurs naturally as the mineral zincite, has been well-studied in biomedicine, including drug or gene delivery, antimicrobial therapy and biosensors, due to its huge amount of storage and simple processing technology (Liu et al., 2016; Wang et al., 2017). Emerging of nanotechnology endow some positive therapeutic effects of ZnO in the form of nanoparticles (NPs), which has the capability to improve the reconstruction and repair of injured neural tissue (Pan et al., 2020). For example, ZnO NPs have been reported to show neurogenic and neuroprotective properties, thereby improving the synaptic connectivity/synaptic plasticity of cortical neurons and finally promoting neurogenesis in a rat model of cerebral ischemia (Barui et al., 2020). However, it remains inconclusive whether ZnO NPs can be used as neuroprotective agents after SCI, and the potential mechanisms of the protective effect still requires further revealed. Thus, taking ZnO NPs as one research objects to develop its positive effect on SCI is urgent and meaningful.

In this study, firstly, we analyzed a total of 48 samples from peripheral blood RNA sequencing (RNA-seq), including healthy group and SCI patients. We determined 10 key post-SCI biomarkers and constructed an artificial neural network (ANN) diagnostic model. We then identified differential alterations after SCI, and indicated that the immune response, oxidative stress, mitochondrial metabolism and cellular apoptosis are the main features. Based on the obtained targets, we elucidated through *in vitro* experiments that ZnO NPs exhibit a neuronal protective effect after SCI, manifested as restrained oxidative stress, enhanced mitochondrial metabolism and decreased cellular apoptosis level. Therein, the PI3K/AKT signaling pathways may play a vital role according to the RNA-seq analysis. We hope these results would provide new insights into the diagnosis and treatment after SCI.

## Methods

### Data collection and processing

The gene expression profile and clinical information in RNA-seq dataset GSE151371 was obtained from the Gene Expression Omnibus database (GEO; https://www.ncbi.nlm.nih.gov/geo/). The RNA-seq data (Raw Count and Fragments Per Kilobase Million [FPKM]) in peripheral blood of healthy control samples (HC, *n* = 10) and acute SCI samples (*n* = 38) were collected and calculated. The GRCh38 v38 version of the gene characteristics for the human genomes was obtained from GENCODE website (https://www.gencodegenes.org/) to identify and annotation mRNA (Frankish et al., 2019). The “Combat” function from R package “sva” was used to remove batch effects and other unwanted variation. Next, the principal component analysis (PCA) based the expression data of samples from GSE151371 cohort was performed to distinguish SCI from HC samples.

### Screening of differentially expressed genes

The R package DESeq2 (v1.36.0) (Love et al., 2014) was utilized to analyze the counts data and detect the DEGs comparing the SCI and HC samples. DESeq2 offer an algorithm for analyzing differential expression levels using RNA-seq raw count data. Genes with adjusted *p* value less than 0.05 and |log2 fold change (FC)| >2 were determined as DEGs. The number, significance and abundance values of DEGs were shown in heatmap and volcano plot using R packages pheatmap and EnhancedVolcano.

### Over-representation enrichment analysis

Over-representation enrichment analysis is a common statistical method to determine whether genes from pre-defined sets are present more than the expected (over-represented). The R package clusterProfiler (Yu et al., 2012) was utilized to conduct enrichment analysis of Gene Ontology (GO) and Kyoto Encyclopedia of genes and Genomes (KEGG) based hypergeometric test. DEGs were used for GO or KEGG enrichment analysis to identify characteristic biological process, function or pathways with *p* value or adjusted *p* value (q-value) <0.05. The dotplot was used to visualize enriched terms and the treeplot function performs hierarchical clustering.

### Gene set variation analysis and single-sample gene set enrichment analysis

To understand biological processes after SCI, we introduce Gene set variation analysis (GSVA) algorithm, which is a methodology based on the enrichment of a specific set of genes that can evaluate alterations in specific pathways activity. The R package GSVA (Hänzelmann et al., 2013) was used to conduct GSVA analysis. A series of key gene signatures from the GO database and KEGG data were collected as background gene sets, mainly including metabolism-related processes, hallmark gene signatures. Next, we analyzed the differential pathways between SCI and healthy groups by R package limma (Ritchie et al., 2015) with |(log2FC)| = 0.2 and adjusted *p* value = 0.05 as the difference threshold.

The single sample gene set enrichment analysis (ssGSEA) algorithm was employed to measure relative abundance of 23 immune cell categories in the immune microenvironment. In the ssGSEA analysis, the relative abundance of every immune cell category is quantified as a fraction score and is standardized to a uniform distribution from 0 to 1. The Wilcoxon rank-sum test was applied to identify the differential levels of each type of immune cells between SCI and healthy groups. The R package corrplot was employed to visualize the correlations among differential immune cells based on Pearson’s correlation analysis.

### Selection of key DEGs using random forest algorithm

The R package randomForest was adopted to establish a random forest model based on DEGs. The prediction error rate on the out-of-bag portion of the data was recorded. The best trees produced by the random forest algorithm is defined by the minimal prediction error rate. Finally, a random forest classifier was built and an importance feature function was used to extract and select variable importance. The genes with an importance value > 0.5 were selected as the key genes. A heatmap was used to depict the unsupervised clusters of these SCI and healthy sample based on these key genes.

### Construction of an ANN diagnostic model

The R package neuralnet (Fritsch and Guenther, 2010) and NeuralNetTools (Beck, 2018) were used to establish an ANN diagnostic model based on key DEGs. First, data normalizing was conducted and each gene expression is transformed and assigned a value [0,1] in the form of a dummy variable. Next, five hidden layers were set to construct a classifier of SCI using key genes weight indices. Finally, the scores calculated by multiplying each gene weight index by the key gene expression values were used as the classification criteria for SCI and healthy groups in the ANN diagnostic model. To evaluate the accuracy of this ANN diagnostic model, we utilized the R package pROC to calculate the AUC value to observe the model classification performance.

### Primary neuron culture and axon axotomy

The primary cortical neuron was acquired from wild-type Sprague-Dawley E18 embryonic rats by enzymes digestion after cesarean section as we previous reported (Kang et al., 2022). The collected neurons were suspended in Neurobasal medium containing GlutaMAX and B27 supplement (Thermo Fisher, United States), and seeded in the left chamber of poly-D-lysine-coated (0.1 mg/ml, Solarbio, China) microfluidics chips (Xona, United States) with 450 μm microgroove barrier. After axons were fully extended into the right chambers, axotomy was performed at the right chambers using a sterilized pipette connected to laboratory vacuum. Aspiration was performed until the axons in right chambers were emptied (Park et al., 2006).

### Characterization of ZnO NPs

ZnO NPs (No.677450) was obtained from Sigma-Aldrich (United States). TEM (H-7500, Hitachi, Japan) was used to observe the morphology and size of NPs. The hydrodynamic diameter and zeta potential of NPs in PBS was measured using a Zetasizer (Malvern Zetasizer Nano, Malvern Panalytical, United Kingdom). The BET specific surface area was measured using ASAP 2020 Plus (Micromeritics, United States) at 77 K.

### Measurement of mitochondrial membrane potential

After axotomy, neurons were treated with 5 μg/ml ZnO NPs for 24 h before detection of mitochondrial membrane potential (MMP) detection. For MMP detection, cells were labelled via 200 nM TMRE (Thermo Fisher, United States) for 45 min and 100 nM Mitotracker (Thermo Fisher, United States) for 30 min, all processes were handled protect from light in cell incubation. Cellular fluorescence images were obtained with confocal microscope (Carl Zeiss, Germany) and analyzed using ImageJ software.

### Real-time PCR analysis

Total RNA extracted via the RNAiso Plus (TaKaRa, China) were reverse transcribed into cDNA via RT reagent Kit (TaKaRa, China). Gene expression was measured by quantitative real-time PCR with application of SYBR Premix Ex Taq™ II (TaKaRa, China) and LightCycler480 System (Roche, Switzerland). The 2-ΔΔCT method were used to calculate the relative gene expression. Primer sequence was listed in Table 1.

**TABLE 1:** List of primer used in this study.

Target	Forward (5′‐>3′)	Reverse (5′‐>3′)
*β-actin*	TAC​AGC​TTC​ACC​ACC​ACA​GC	TCT​CCA​GGG​AGG​AAG​AGG​AT
*Bax*	CTG​GAT​CCA​AGA​CCA​GGG​TG	CCT​TTC​CCC​TTC​CCC​CAT​TC
*Bcl-2*	GAA​CTG​GGG​GAG​GAT​TGT​GG	GCA​TGC​TGG​GGC​CAT​ATA​GT
*SOD1*	ACC​ACT​GCA​GGA​CCT​CAT​TTT	CTT​CAT​TTC​CAC​CTT​TGC​CCA
*SOD2*	GCA​CCA​CAG​CAA​GCA​CCA​C	AAC​TCC​CCT​TTG​GGT​TCT​CC
*IL-4*	GTA​CCG​GGA​ACG​GTA​TCC​AC	GTG​AGT​TCA​GAC​CGC​TGA​CA
*IL-10*	AGA​AGC​ATG​GCC​CAG​AAA​TCA	GGCCTTGTAGACACCTTGGT
*IL-1β*	CTC​GTG​CTG​TCG​GAC​CCA​T	CAG​GCT​TGT​GCT​CTG​CTT​GTG​A
*TNF-α*	CCT​GTA​GCC​CAC​GTC​GTA​G	GGG​AGT​AGA​CAA​GGT​ACA​ACC​C

### mRNA sequencing

Total RNA extracted with Trizol reagent was used as the starting RNA for library construction. mRNA with polyA tails was identified by Oligo (dT) beads. Then, mRNA was fragmented by incubation with Magnesium ions in NEB Fragmentation Buffer. Next, cDNAs are synthesized using the mRNA as a template. The purified double-stranded cDNA is further processed to screen out cDNAs of about 250-300 bp. cDNAs were amplified by PCR and purified again to obtain a final library. After library construction, the libraries are quantified and quantified to 1.5 ng/μl. The libraries are pooled at the defined concentration and the preset data size, and then sequenced in Illumina to generate 150 bp paired-end reads. The sequenced fragment information was identified and converted into reads data, which were stored in fastq format. The reads are then matched to the reference genome through data quality control and sequence alignment, and finally raw counts data are calculated using featureCounts software.

### Statistical analysis

Data were expressed as means ± standard error of mean, and a value of *p* < 0.05 was considered significant. Comparison of two means was performed by using a two-tailed Student *t* test. All statistical analyses in this study were conducted using R (version 4.2.0) or SPSS software (version 22.0).

## Results

### Identification of DEGs and enrichment analysis from peripheral blood samples

**FIGURE 1:**
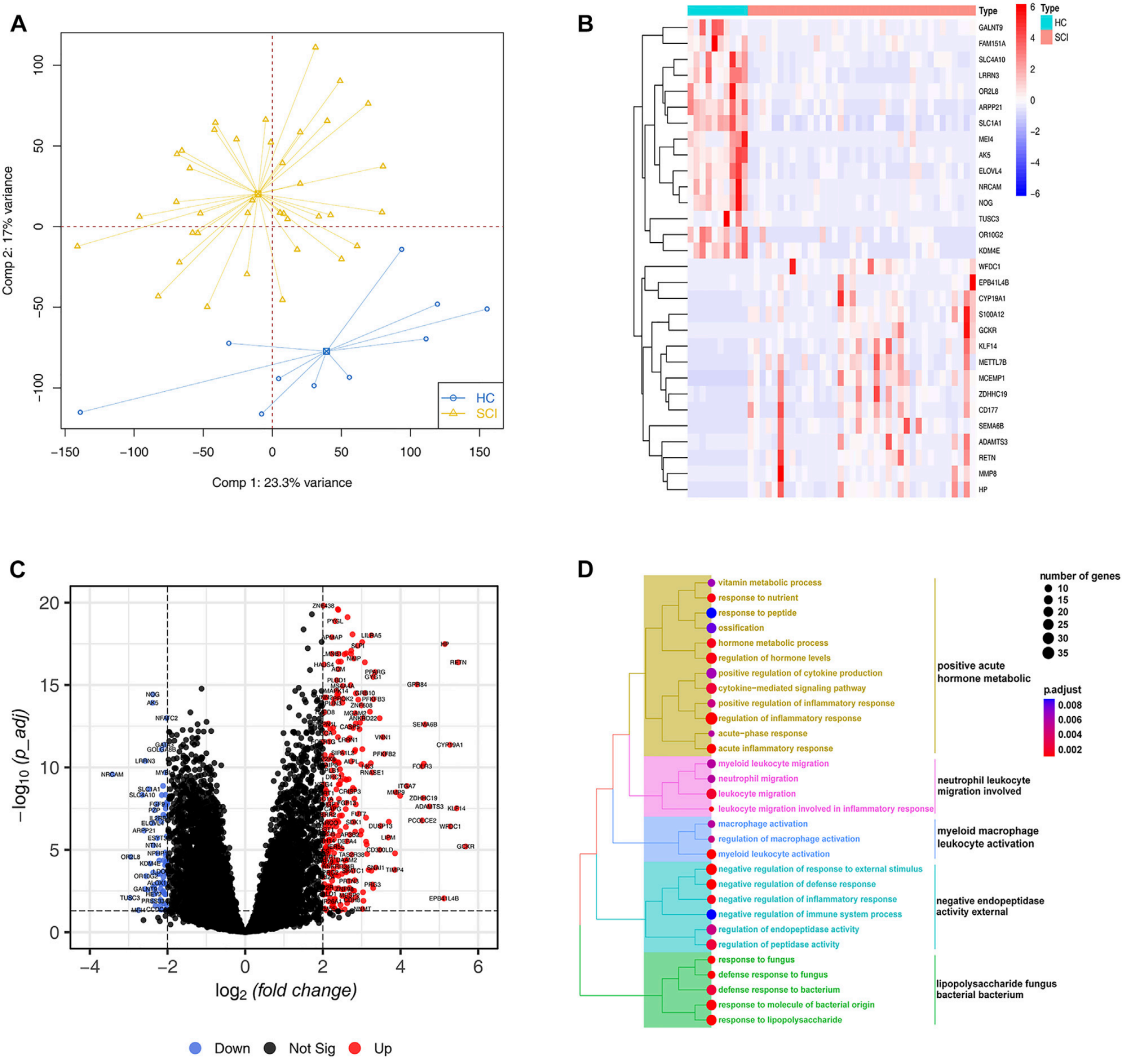
Differential expression and enrichment analysis. **(A)** Principal component analysis for the expression profiles to distinguish spinal cord injury (SCI) from healthy controls (HC) in GSE151371 cohort. Two subgroups are clearly distinguished based on the mRNA expression levels. SCI samples are marked with yellow and healthy controls marked with blue **(B)** Heatmap plot of top 30 differentially expressed genes (DEGs) from the comparison of SCI with HC filtered by the fold change. High expression is indicated in red and low expression is indicated in blue. **(C)** The volcano plot shows the number and distribution of all genes. Each dot represents one gene with differential up-regulated in SCI (red), differential down-regulated (blue) or no significant difference (black). **(D)** The treeplot shows GO and KEGG enrichment summary of DEGs. GO terms and KEGG pathways categories are grouped according to functional terms.

To gain a comprehensive understanding of the potential differences in biological processes after acute SCI versus the healthy group, we first performed a PCA analysis and found SCI samples were well distinguished based on the gene expression profiles (Figure 1A). Next, we used the annotation information from GENCODE to extract the mRNA levels expression data matrix. 374 DEGs were screened, in which 294 were upregulated genes and 80 were downregulated genes in SCI group, compared to healthy group. The expression levels of the top 30 DEGs sorted by their log2FC in samples is showed in Figure 1B. Furthermore, a volcano plot showed the distribution of these DEGs, including their adjusted *p* value and log2FC (Figure 1C). To further characterize the biological significance among these DEGs, GO enrichment analysis indicating immune response and metabolic related terms were enriched, such as neutrophil leukocyte migration involved and myeloid macrophage leukocyte activation (Figure 1D).

### Random forest screening and construction of the ANN model for SCI

**FIGURE 2:**
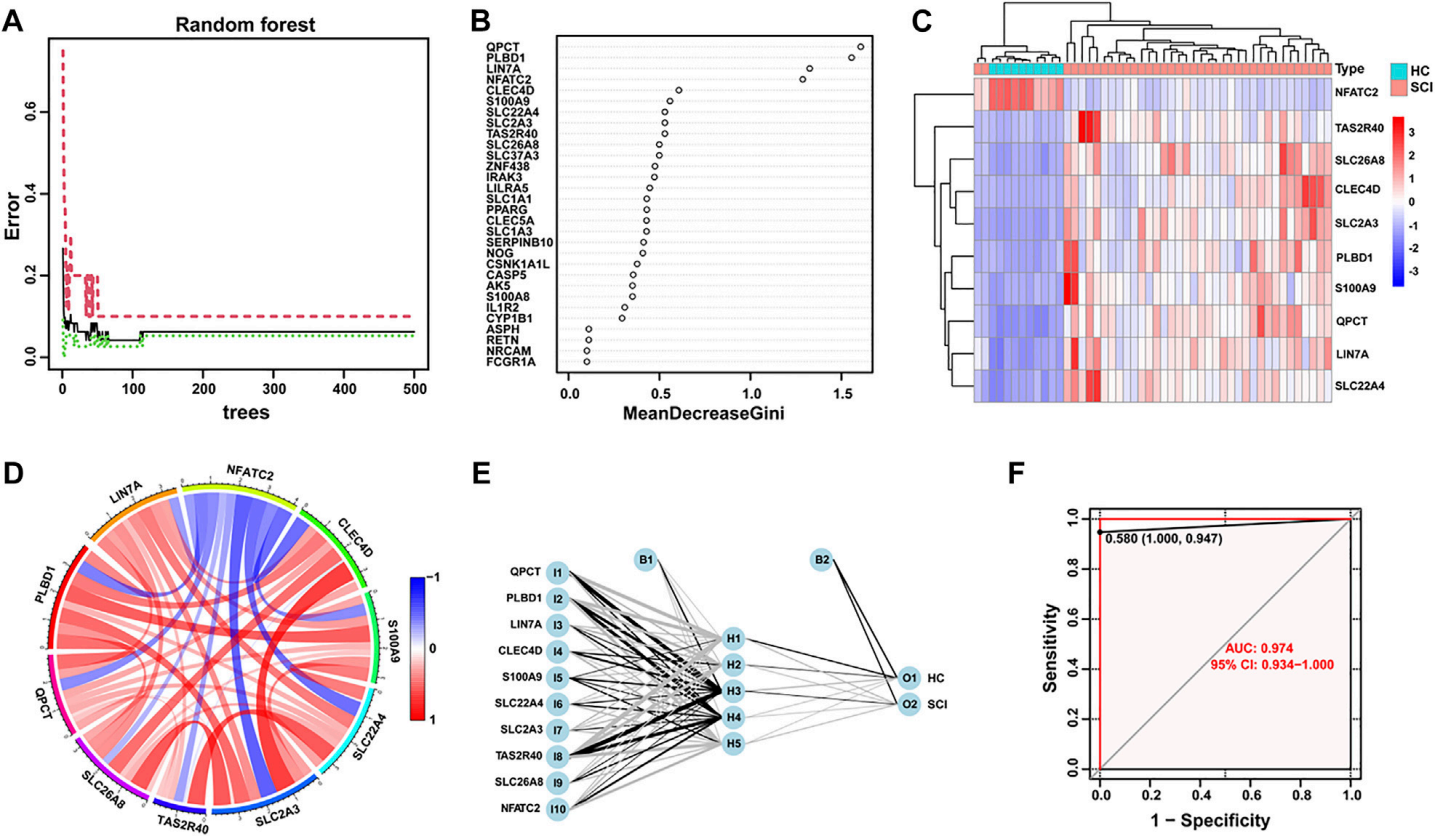
Random forest screening of genes and construction of neural networks. **(A)** The changes of error rate with the different number of decision trees in random forest. The number of trees is shown in x-axis, the error rate is calculated in the y-axis. **(B)** Importance levels of genes were calculated and ranked using Gini coefficient method in random forest algorithm. The x-axis indicates the Gini coefficient, and the y-axis shows the gene symbol. **(C)** A heatmap with unsupervised clustering shows SCI and healthy control samples could be well distinguished by 10 key DEGs. Red color represents genes with high expression in the SCI group, blue color represents genes with low expression. **(D)** The Circos plot shows the correlation of the expression levels of the 10 key DEGs. The colors correspond to the direction of correlation, with red indicating positive relationships and blue indicating negative correlations. **(E)** Visualization of a neural network model, while 10 key DEGs are used as input layer. **(F)** The ROC curve reflects the accuracy of the neural network diagnostic model.

To screen for key genes, 374 DEGs were inputted into the random forest model. We conducted a random forest classification for gene variables and calculated the prediction error rate of the model. Figure 2A depicts the error rate of the model as the number of trees changes. We chose the number of trees with the lowest error rate, which indicates that the model error is relatively stable. The variable importance based on Gini coefficient method was also determined. We next selected top 10 DEGs based on importance function as the key genes. Figure 2B displayed that these 10 variables and their importance level, *QPCT*, *PLBD1*, *LIN7A*, *NFATC2*, *CLEC4D*, *S100A9*, *SLC22A4*, *SLC2A3*, *TAS2R40* and *SLC26A8*. Based on these gene expression data of 10 key variables, we depicted a heatmap and performed unsupervised clustering. As shown in Figure 2C, these genes could be clearly separated between the SCI and HC samples. Importantly, only the gene NFATC2 showed high expression level in the healthy group and low expression level in the SCI group. On the contrary, *TAS2R40*, *SLC26A8*, *CLEC4D*, SL*C2A3*, *PLBD1*, *S100A9*, *QPCT*, *LIN7A* and *SLC22A4* genes showed low expression level in the SCI group and high expression level in the healthy group. The correlations and interactions of genes expression between the 10 key genes also showed that only NFATC2 showed negative correlation with other genes (Figure 2D).

Next, we developed an ANN model based on the above 10 key DEGs to help diagnose SCI in the acute phase. The pre-processing of the expression data was performed and 5 hidden layers was set up. We then used the candidate genes as input layers and completed the construction of the ANN diagnostic model (Figure 2E). The connections weights between each layer in the neural network model are summarized in Supplementary Table 1. Finally, the receiver operating characteristic (ROC) curve based on bootstrap method was used to determine the diagnostic model performance. The areas under the ROC curves (AUC) were near to 1 (average AUC = 0.974, Figure 2F), which indicates high accuracy for this diagnostic model.

### Understanding the alterations in biological processes after SCI

**FIGURE 3:**
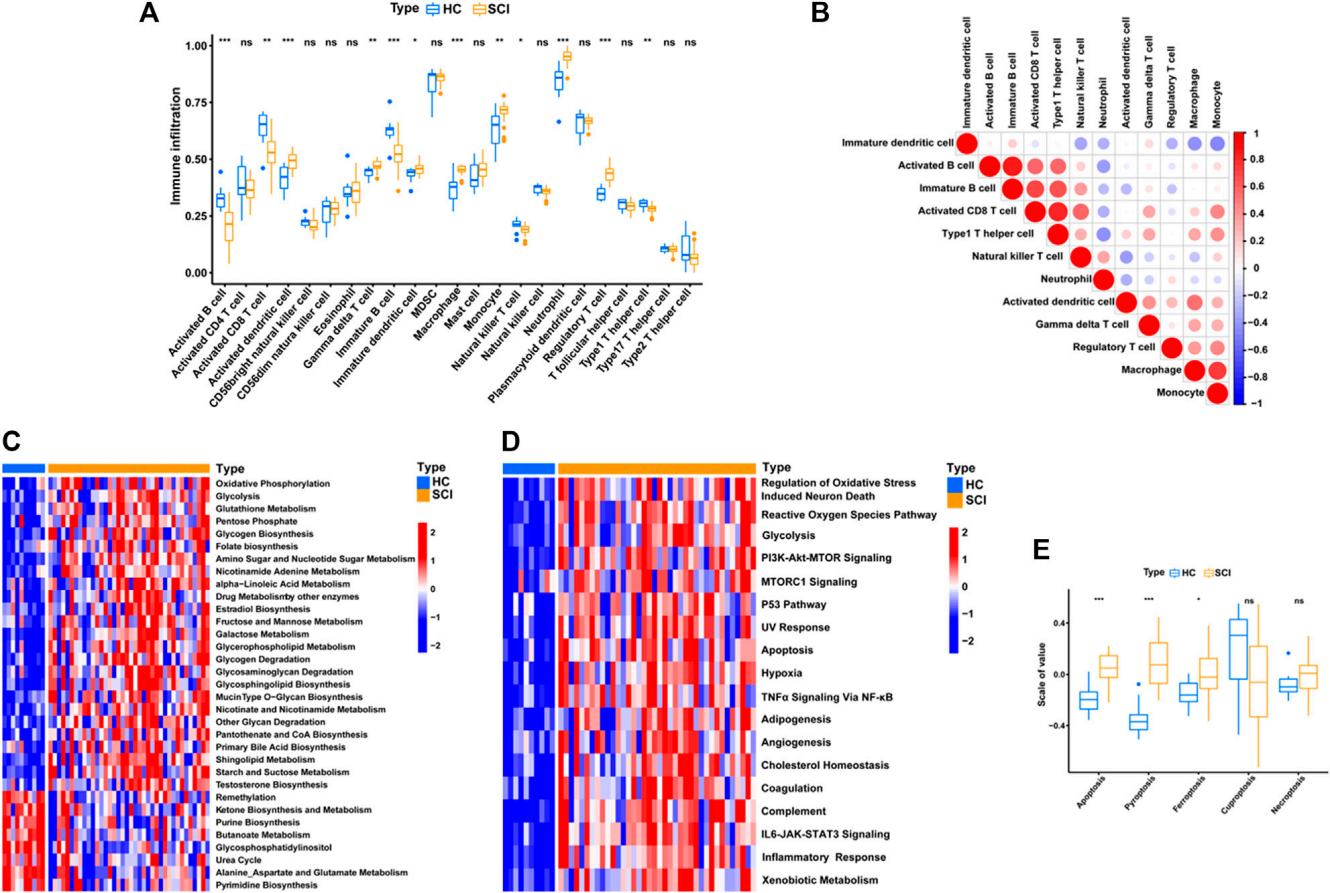
Identify immunological, metabolic, and biological features of SCI. **(A)** Box plot shows the abundance and difference of 23 immune cell types between SCI and HC groups. *P* values were showed as: ns, not significant; *p < 0.05; **p < 0.01; ***p < 0.001. **(B)** Correlation matrix for all 12 differential immune cell proportions. Negative correlations are marked in blue and positive correlations are marked in red. **(C–D)** Heatmap of GSVA results showing GSVA scores of the metabolic pathways and hallmark gene set. **(E)** This boxplot shows an overview of the levels of cell death-related pathway activation between the SCI and HC groups, including ferroptosis, pyroptosis, apoptosis, cuproptosis and necroptosis. *P* values were showed as: ns, not significant; *p < 0.05; ***p < 0.001.

After SCI, various immune cells were recruited to the lesion site through blood approach and immune response would be activated, which implied that the transcriptional alterations in peripheral blood could partly reflects the biological situation of lesion site after SCI. To further explore the biological behaviors after SCI, we performed the ssGSEA enrichment analysis to analysis immune cell infiltration level. We quantified the relative abundance of 23 immune cell types and found that 12 of them were significantly different, including activated B cell, activated CD8^+^ T cell, activated dendritic cell, gamma delta T cell, immature B cell, immature dendritic cell, macrophage, monocyte, natural killer T cell, neutrophil, regulatory T cell and type 1 T helper cell (Figure 3A). A correlation circos plot of the differential immune cells revealed that immature B cells were positively related with the activated B cells (*r* = 0.87) and type 1 T helper cells (*r* = 0.66), activated CD8^+^ T cells were positively related with type 1 T helper cells (*r* = 0.82) and immature B cell (*r* = 0.69), macrophage were positively related with monocyte (*r* = 0.72), whereas immature dendritic cell was negatively related to monocyte (*r* = -0.47) (Figure 3B).

The metabolic related biological processes between SCI and healthy group were also explored by using GSVA enrichment analysis. As shown in Figure 3C, oxidative phosphorylation, glycolysis, folate biosynthesis, amino sugar and nucleotide sugar metabolism, nicotinamide adenine metabolism and glycerophospholipid metabolism were remarkably up-regulated in the SCI group, whereas the purine biosynthesis and butanoate metabolism were remarkably down-regulated. Intriguingly, most of these biological processes are involved with the mitochondrion homeostasis, such as oxidative phosphorylation, glycolysis, which implied that mitochondrial metabolism was one main features after SCI. The literature has shown that for the injured axon to successfully regenerate after SCI, it is necessary to reseal the damaged terminal, rebuild the cytoskeleton, synthesize and transport building materials, assemble axonal components, and form growth cones, all of which require a large amount of energy in the form of ATP, which is mainly supplied by mitochondria in neurons (Han et al., 2020). Similarly, these immune cells in peripheral blood should also require enhanced energy metabolism to guarantee the feedback immune response at SCI lesion site. Thus, mitochondrial metabolism is urgently needed to sustain energy supply after SCI, as a result inducing upregulation of these mitochondrial metabolism related biological processes.

Next, we quantified and analyzed the levels of some classical signaling pathways and found that the SCI group had higher levels of oxidative stress induced neuronal death, PI3K-Akt-mTOR signaling and inflammatory response (Figure 3D). Given the systemic stress and injury environment after SCI, we further analyzed the cell death patterns and found that apoptosis, ferroptosis and pyroptosis were significantly more activated in SCI than in the healthy group (Figure 3E).

To conclude, there is a significant difference in immune cell infiltration and metabolic related processes between patients with SCI and healthy group. The above results suggest that the recruitment and activation of immune cells in the SCI group were significantly enhanced, then these activated immune cells are not only responsible for responding to damage and generating immune responses (eg., inflammatory response), but also could produce “respiratory burst effect” and generate superoxide radicals at the same time. Accordingly, we revealed that the oxidative stress was also significantly up-regulated in SCI groups, and was close related to the fate of injured neurons.

### ZnO NPs exhibit a protective role on neurons after SCI

For neurons, the mitochondrion could be quickly damaged after SCI, where the mitochondrion-mediated energy metabolism could be reduced potently at the lesion site (Zhou et al., 2016). Then, the damaged mitochondria-released reactive oxygen species (ROS) would further impair the adjacent mitochondria and following lead to more mitochondrial ROS generation, a pathway known as the “ROS-induced ROS generation”. This is one main origins of the oxidative stress in injured neurons, later lead to cell death. Furthermore, the damaged axon debris could activate the immune response and recruit immune cells systemically. This cell-mediated immunity contributed to creating an oxidative environment, which return exacerbate neuron death as well.

**FIGURE 4:**
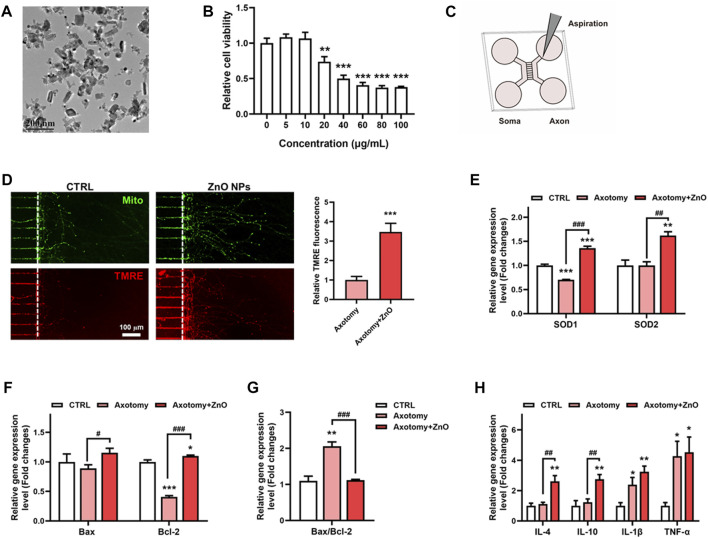
ZnO NPs exhibit a protective role on neurons after axotomy. **(A)** The morphology and size of ZnO NPs were characterized using TEM. **(B)** The cell viability assay of neurons after ZnO NPs treatment with various concentrations for 24 h. **(C)** Schematic diagram of constructing an *in vitro* neuron axotomy model using microfluidic chip. **(D)** After aspiration, ZnO NPs were treated for 24 h, MitoTracker and TMRE dyes were used to label mitochondria and functional mitochondria respectively, and the TMRE fluorescence intensity in axon regeneration terminals was statistically analyzed. **(E)** qRT-PCR data of SOD1 and SOD2 in neurons treated axotomy with or without ZnO NPs treatment for 24 h; changes in the gene expression levels were quantified after normalization against β-actin. **(F–G)** qRT-PCR data of Bax and Bcl-2 in neurons treated axotomy with or without ZnO NPs treatment for 24 h; changes in the gene expression levels were quantified after normalization against β-actin, and the Bax/Bcl-2 ratio was calculated and statistically analyzed as well. **(H)** qRT-PCR data of IL-4, IL-10, IL-1β and TNFα in neurons treated axotomy with or without ZnO NPs treatment for 24 h; changes in the gene expression levels were quantified after normalization against β-actin. *p < 0.05; **p < 0.01; ***p < 0.001 compared with the control group. ^#^ *p* < 0.05; ^##^ *p* < 0.01; ^###^ *p* < 0.001 compared with the axotomy group.

ZnO NPs, as one natural agents, has become extensively applied in biomedicine. In this study, we investigate whether ZnO NPs could exert some positive effects on SCI models. Firstly, we detected the characterizations of ZnO NPs. The TEM data revealed that the ZnO NPs were rod shaped, and their length was 55.3 ± 23.1 nm and width was 31.3 ± 10.2 nm (Figure 4A), and the size of the aggregates in PBS was 143.4 nm. The BET analysis showed that the surface area of the ZnO NPs was 28.3 m^2^/g. The zeta-potential of the ZnO NPs was -14.4 mV (Table 2). In order to evaluate the cell biocompatibility of ZnO NPs, we carried out cell viability assay. Our data showed that when the concentration was above 20 μg/ml, ZnO NPs exhibit a potent toxic effect with concentration dependent manner (Figure 4B). Interestingly, our previous study has indicated that the ZnO NPs with concentration of 5 μg/ml possess positive effects, which could promote the cell proliferation via cellular kinases modulated cell cycle pathway (Jia et al., 2017). Thus, in view of above results, 5 μg/ml with non-toxic effects were applied as the main concentration in this study.

**TABLE 2:** Characterization of ZnO NPs.

**Average size (TEM)**			**Particle size (DLS)**	**Zeta potential**	**BET surface area**	**Total pore volume**	**Average pore diameter**
**Length**	**Width**						
55.3 ± 23.1 nm		31.3 ± 10.2 nm	143.4 nm	−14.4 mV	28.3 m²/g	0.1 cm³/g	14.4 nm

Then, we applied microfluidic culture devices to construct one *in vitro* SCI model, in which neuronal cell bodies and dendrites are restrained to the soma chamber while their axons grow into the axon terminal chamber through long (450 µm in length) microgrooves. After axons were fully extended into the axon terminal chamber, axotomy of axons via aspiration was performed (Figures 4C,D). We first detected the mitochondrial hemostasis via measuring the mitochondrial membrane potential. With all mitochondria labeled via MitoTracker, the functional mitochondria were labelled via TMRE, one fluorochrome could label the mitochondrion with normal mitochondrial membrane potential, simultaneously. Interestingly, our data showed that after axotomy, the axon regeneration was potently enhanced after ZnO NPs treatment when compared with the axotomy group. Moreover, the TMRE fluorochrome intensity was also boosted under treatment of ZnO NPs, which implied an enhanced energy supplement (Figures 4C,D). These data indicated the ZnO NPs played a helpful role on the mitochondrial metabolism after neuronal injury, and could potently alleviate the SCI-induced oxidative stress due to a quick-restored mitochondria homeostasis.

In order to unveil the alteration in oxidative stress of SCI status after ZnO NPs treatment, we detected the gene expression levels of SOD1 and SOD2, two main oxidoreductase which could eliminate the excess superoxide radicals. Our data showed that after axotomy, the expression level of *SOD1* slightly decreased but have no significantly change in expression level of *SOD2*. ZnO NPs treatment could potently increase the expression level of these two proteins after axotomy, and ever higher than the expression level in the control group (Figure 4E). As oxidative stress has been reported as one main factor for leading to cell apoptosis after SCI, we investigated the apoptosis involved gene levels with or without ZnO NPs treatment. Mitochondrial stress-induced apoptosis is mediated via various proteins, therein, Bcl2 and Bcl2-associated X (Bax) are the main ones, which exhibited an anti-apoptosis and pro-apoptosis effects, respectively. After axotomy, ZnO NPs could increase the gene expression level of both *Bax* and *Bcl2*. Importantly, a significant increase of *Bax*/*Bcl2* ratio were observed in axotomy group but the ZnO NPs treatment could successfully rescue the *Bax*/*Bcl2* ratio, which implied a decreased apoptosis level (Figures 4F,G).

Meanwhile, as the inflammatory effects are one main features of SCI, we also developed the inflammatory status via detecting gene expression levels of inflammatory cytokines, including anti-inflammatory cytokines like IL-4 and IL-10, and pro-inflammatory cytokines like IL-1β and TNF-α (Figure 4H). Our data showed that, after axotomy, the expression level of pro-inflammatory cytokines IL-1β and TNF-α were all potently increased, but no significantly changes were observed for the anti-inflammatory cytokines IL-4 and IL-10. Importantly, ZnO NPs treatment could enhance the gene expression of IL-4 and IL-10, while showed few changes on the gene expression of IL-1β and TNF-α. We suggested that the ZnO NPs probably exhibit anti-inflammatory effect via up-regulating the gene expression of anti-inflammatory cytokines, which was in accordance to the previous reported data (Lin et al., 2021).

### PI3K/Akt signaling pathway play a vital role in ZnO NPs’ protective effects after SCI

**FIGURE 5:**
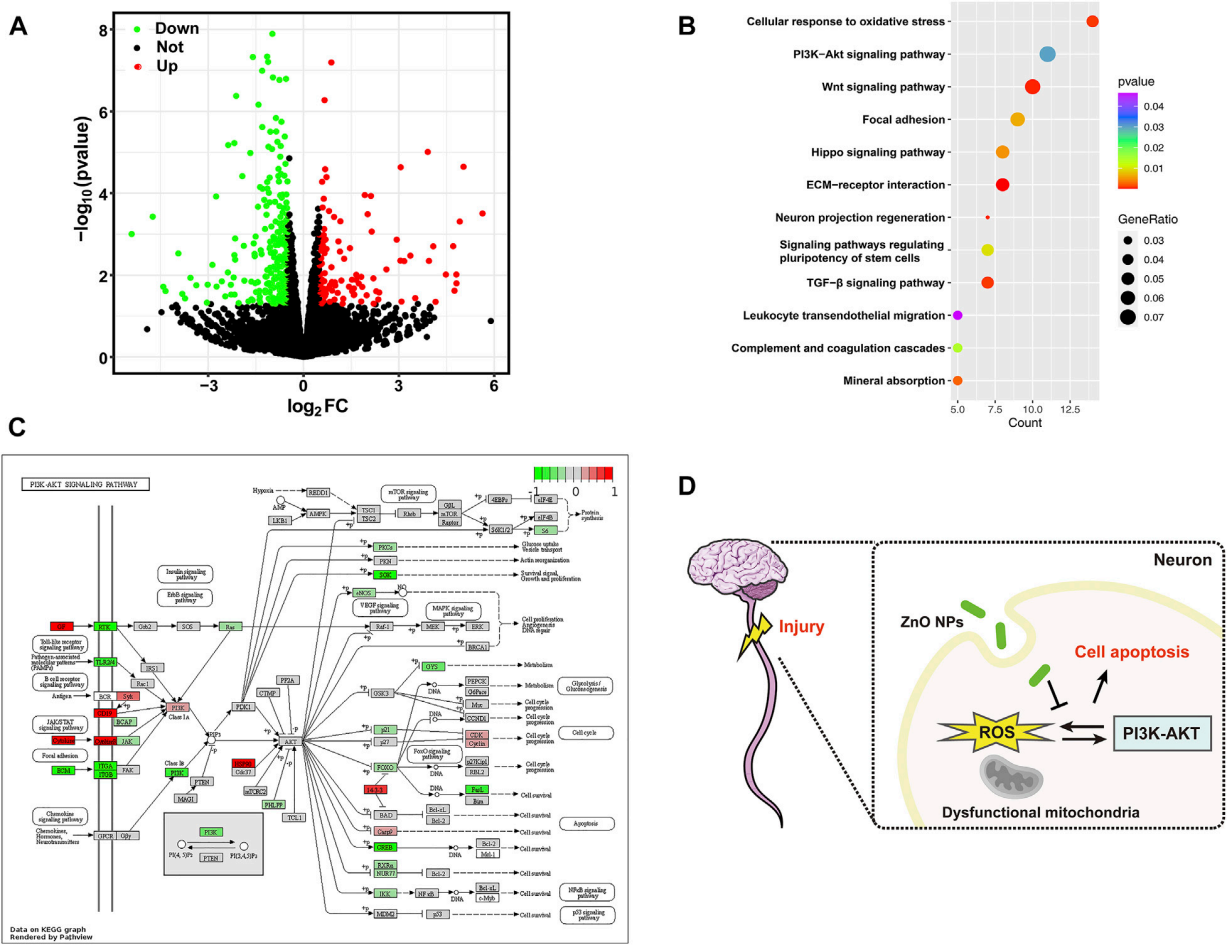
**(A)** Volcano plot depicting an overview of the differential expression of all genes. **(B)** The bubble plot shows significantly enriched GO terms and KEGG pathways with *p* value < 0.05. **(C)** A visualized DEGs (comparison ZnO treatment group and axotomy group) expression map for PI3K-Akt signaling pathway from KEGG database. Red and green indicate genes activated or suppressed in this pathway, respectively. **(D)** Schematic illustration of the study. ZnO NPs protect neuronal cells from apoptosis after SCI by inhibiting ROS levels. The PI3K-Akt pathway may be a key mechanism involved.

To further explore the potential mechanisms after treatment with ZnO NPs, we treated axotomized neurons with ZnO NPs (*n* = 3) or controls (*n* = 3) and performed an RNA-seq on these 6 samples. Figure 5A shows 376 DEGs were identified, including 122 up-regulated genes and 254 down-regulated genes. We then performed an enrichment analysis on these DEGs to determine the potential biological changes related to their alterations. As shown in Figure 5B, the neuron projection regeneration, cellular response to oxidative stress and PI3K-Akt signaling pathway were significantly enriched. To further clarify the potential changes of PI3K-Akt signaling pathway after ZnO NPs treatment, we mapped an overview of the gene changes under the pathway by pathview method (Figure 5C). We found that PI3K family genes associated with chemokine signaling, hormones, and neurotransmitter were significantly downregulated, suggesting a downregulation trend of PI3K-Akt signaling pathway. Finally, we summarize the potential mechanisms by which ZnO NPs act as a protective agent for axotomized neurons (Figure 5D).

## Discussion

SCI causes tremendous physical and psychological damage to patients, and the effective treatment still remain a major challenge. A great deal of current research has focused on the microenvironment at the lesion site in an attempt to provide biological basis for treatment, but little attention has been paid to the patient’s whole-body condition. In addition to the need to develop therapeutic strategies, there are still exist some challenges in the diagnosis of SCI patients. MRI is often used in the clinical diagnosis of SCI patients to provide information about the severity of the injury and the position of spinal cord damage. However, it is not always available in some patients, especially when they are unresponsive or obtunded and those with penetrating metal injuries (Elizei and Kwon, 2017). Previous studies have pointed out the use of some biomarkers in peripheral blood can be a key and stable predictor of acute SCI (Tigchelaar et al., 2019; Schading et al., 2021). In this study, we analyzed a peripheral blood RNA-seq cohort after SCI (GSE151371), including an RNA expression profile and clinical information. Among the identified DEGs, almost 80% of the genes were significantly upregulated in SCI, which heralds a dramatic change in the microenvironment-related biological processes. In order to fully exploit the value of SCI peripheral blood RNA-Seq samples to provide some profitable biomarkers for diagnosis of SCI, we developed an accurate and accessible diagnostic prediction model. Based on the variable importance algorithm of the random forest classifier, we downscaled the number of DEGs to obtain key candidate DEGs as biomarkers of SCI. We finally selected 10 key DEGs as gene signatures, and constructed a SCI neural network diagnostic model based on these candidates. We evaluated the classification performance of the ANN model by ROC curve. The AUC value in the ROC curve shows that the ANN model has excellent diagnostic ability, that is, high classification accuracy.

Of these 10 gene signatures, it is worth noting that the mRNA expression levels of S100 calcium-binding protein A9 (S100A9) were increased in the injured spinal cord. S100A9 protein encoded by this gene is a member of the S100 family of proteins, which are usually found in immune cells, and could be regarded as an inflammatory signal alarm (Pruenster et al., 2016). The use of S100A9 inhibitors at the time of early SCI was demonstrated to remarkably reduce the inflammatory response and inhibit the apoptosis of injured neurons (Sun et al., 2022). Besides, we identified NFATC2 as another potential biomarker. This protein plays a central role in inducing gene transcription during the immune response and axons growth in an *in vitro* model treated with neurotrophins (O'Donovan, 2016). A previous investigation showed that significant changes in the mRNA levels of NFATC2 in the spinal cord were not observed between days 3 and 14 after injury (Cai et al., 2013). However, our analysis showed that NFATC2 was significantly downregulated in the SCI group, suggesting that it could serve as a specific biomarker in the early or acute phase of SCI.

To explore the potential therapeutic targets of SCI from the RNA-seq cohort, we next explored the biological processes changes of the microenvironment within the blood. GO enrichment analysis revealed that DEGs were significantly enriched to immune response and metabolism related terms. At the immune level, we quantified the level of 23 immune cell infiltrations after the onset of SCI using the ssGSEA algorithm and found that 7 key immune cells were significantly increased in the SCI group. These results suggest that these immune cells could react to the injury and may play an important role in the SCI-related immune response. The immune cell correlation analysis also provides a reference for future studies on immune response to SCI. Meanwhile, some metabolic pathway, such as the glutathione metabolism, oxidative phosphorylation and glycogen biosynthesis were significantly upregulated in the SCI group, which provide a reference for metabolic support therapy and nutrient intake for acute SCI (Farkas et al., 2021; Li et al., 2021). Importantly, these processes were all mitochondrion involved and reflect changes on mitochondrial metabolism after injury (Rabchevsky et al., 2020; Slater et al., 2022). The GSVA method also confirm the alterations in mitochondrial metabolism-involved pathways alterations, including enrichment of oxidative stress induced neuron death, PI3K/Akt/MTOR signaling and apoptosis. In total, these analyses indicate the immune response, oxidative stress, mitochondrial metabolism and cellular apoptosis are the main features after SCI.

In a normal physiological state, the redox balance is maintained by timely removal of necrotic cells and metabolic waste. However, oxidative stress occurs due to this balance is upset in an injured state (Checa and Aran, 2020). Injury-initiated mitochondrial damage can induce excessive oxidative stress that causes secondary damage to injured neurons, manifested as the excessive ROS generation, energy metabolism disturbance and apoptosis-involved signaling pathway activation (Xu et al., 2005; Visavadiya et al., 2016). Moreover, the results of our analysis suggest that oxidative stress in peripheral blood is hyperactivated after SCI. In SCI, some monocytes differentiate into activated macrophages in the injured spinal cord, among which M1-activated macrophages dominate the lesion site at early time and initiate secondary injury by secreting enzymes and pro-inflammatory cytokines. At the same time, these activated immune cells also generated ROS by increasing NADPH oxidase activity, and these infiltrated ROS promoted the apoptosis of neurons and glial cells within 24 h (Tran et al., 2018). These evidences suggested that the oxidative stress may be a common and consistent biological feature of the systemic circulation and in the injury sites for the patient with SCI. Previous studies have reported that eliminating ROS protects neurons after injury and promotes nerve regeneration (Jia et al., 2012; Wang et al., 2020), suggesting that to explore more and better potential natural products with antioxidant propertie could be an effective therapeutic manner to promote SCI repair.

Due to the nanometer size of NMs, they can not only easily carry drugs or molecules across the blood-spinal cord barrier to target tissues and cells, but also directly modulate the biological effects related to axonal regeneration, ultimately exerting therapeutic effects on SCI (Kim et al., 2017; Domínguez-Bajo et al., 2019). However, many studies have shown that NMs play an important role in nerve regeneration after SCI, mostly in the form of scaffolds to load therapeutic drugs or provide regeneration space (Zhao et al., 2018; Woods et al., 2022), and few people pay attention to unveil the intrinsic role of NMs in nerve regeneration. In this study, ZnO NPs function both as a natural product and as a nanoparticle. Our recent review summarized and emphasized the potential therapeutic role of nanomaterials in neurological diseases (Jiang et al., 2022). Also, a previous study reported that nanoparticles with antioxidant enzymes were injected into the circulation to reduce systemic inflammation levels and promote SCI lesion repair (Andrabi et al., 2020). However, the specific protective role of ZnO NPs in the treatment of SCI remains unclear. Therefore, we established an *in vitro* model of axotomy and treated with ZnO NPs. The dose of ZnO NPs we applied was a biological safe according to cell viability assay data and our previous studies (Jia et al., 2017; Liu et al., 2019). The data showed that the apoptosis process of neurons was inhibited after treatment with ZnO NPs. In addition to this, ZnO NPs promoted the restoration of MMP and mitigated mitochondrial dysfunction. Meanwhile, the inflammatory effects would also be inhibited mainly due to the promotion effects on anti-inflammatory cytokines gene expression.

Therefore, we confirmed that ZnO NPs could protect damaged neurons from apoptosis after SCI. To further explore the underlying mechanism, we performed RNA-seq and found that the PI3K/Akt signaling may be a key regulatory pathway. Previous studies have reported that the PI3K/Akt signaling could positively promote ROS generation via regulation of NADPH oxidases or mitochondrial processes (Chatterjee et al., 2012; Koundouros and Poulogiannis, 2018). Meanwhile, other literatures also indicated that ROS generation could directly activate the PI3K/Akt signaling pathway (Chen et al., 2017; Zhang et al., 2021). Our study suggests that the PI3K/Akt signaling may be the potential mechanism by which ZnO NPs reduce the level of oxidative stress. Therein, the primary injury-induced mitochondrial damage and corresponding mitochondrial ROS generation would activate the PI3K/Akt signaling pathways, which further aggravate the mitochondrial ROS generation as a cycle, and finally lead to cell apoptosis.

In summary, we mainly revealed the transcriptomic situation of peripheral blood after acute SCI, elucidated the protective effect of ZnO NPs in SCI and developed a diagnostic prediction model in the study, which could provide references for the diagnosis and treatment of SCI in the future.

## Data Availability

The datasets presented in this study can be found in online repositories. The names of the repository/repositories and accession number(s) can be found below: https://www.ncbi.nlm.nih.gov/, PRJNA855585.
